# Downregulation of Heparanase Expression Results in Suppression of Invasion, Migration, and Adhesion Abilities of Hepatocellular Carcinoma Cells

**DOI:** 10.1155/2015/241983

**Published:** 2015-12-29

**Authors:** Xiao-Peng Chen, Jun-Sheng Luo, Ye Tian, Chen-Lin Nie, Wei Cui, Wei-Dong Zhang

**Affiliations:** Department of Hepatobiliary Surgery, Affiliated Yijishan Hospital of Wannan Medical College, Wuhu 241001, China

## Abstract

*Objective.* Heparanase (HPSE) is high-expressed in most malignant tumors including hepatocellular carcinoma (HCC) and promotes cancer cell invasion and migration. The aim of the study is to explore whether HPSE enhances adhesion in metastasis of HCC cells.* Methods*. HPSE expressions in human HCC cells were measured with real-time RT-PCR and Western blot analysis. Four recombinant miRNA vectors pcDNATM6.2-GW/EmGFP-miR-HPSE (pmiR-HPSE) were transfected into HCCLM3 cell. HPSE expression in transfected cell was measured. The cell invasion, migration, and adhesion abilities were detected, respectively.* Results*. Both HPSE mRNA and protein relative expression levels were higher in HepG2, BEL-7402, and HCCLM3 cells than those in normal hepatocyte (*P* < 0.05). HPSE showed highest expression level in HCCLM3 cell (*P* < 0.05). Transfection efficiencies of four miRNA vectors were 75%–85%. The recombinant vectors significantly decreased HPSE expression in transfected HCCLM3 cells (*P* < 0.01), and pmiR-HPSE-1 showed best interference effect (*P* < 0.05). pmiR-HPSE-1 significantly decreased the penetrated and migrating cells numbers and adherence rate of HCCLM3 cells (*P* < 0.05).* Conclusion*. HPSE is a potentiator of cell adhesion in metastasis of HCC.

## 1. Introduction

Globally, hepatocellular carcinoma is the sixth most common cancer and the third most common cause of cancer-related deaths [1]. The high mortality of HCC is mainly due to the occurrence of intrahepatic metastases. Multicentric occurrence of HCC is essentially the result of intrahepatic metastasis [2–4].

Intrahepatic metastasis of HCC is a complex and multistep biological process that includes cancer cell adhesion, invasion, migration, and proliferation. Formation of tumor microembolus in portal vein is one of the crucial links, and HCC cell adhesion is the prerequisites. Cancer cells in blood vessel can activate blood coagulation to induce tumor microembolus through multiple mechanisms [5–7]. But it is still unclear what molecules contribute to cell adhesion and tumor microembolus of HCC, and the precise pathogenesis of intrahepatic metastasis remains to be determined.

Heparanase (HPSE) is an endo-beta-glucuronidase that is capable of cleaving heparan sulfate (HS) side chains of heparan sulfate proteoglycans (HSPGs) on cell surfaces and extracellular matrices (ECM) of basement membrane (BM) and plays critical roles in tumor cell invasion, migration, and angiogenesis by remodeling ECM and delivering some cytokines such as basic fibroblast growth factor (bFGF) and vascular endothelial cell growth factor (VEGF) [8–10]. Increased HPSE expression was found in numerous tumor types and correlates with poor prognosis [11, 12], and downregulation of heparanase expression results in suppression of tumor invasion and migration, especially in HCC cells [13–15]. In recent 15 years, HPSE has become a research hotspot [16, 17]. On the other hand, HPSE may neutralize the anticoagulation properties of heparin and low-molecular-weight heparin and shows procoagulation activity resulting in cancer progression [18–20]. Therefore, HPSE might be a promising target for potential antiadhesive agents [21]. Against this background, we hypothesize that HPSE might play a proadhesive role in adhesion and tumor microembolus of HCC cells.

The aim of this study is to explore whether HPSE enhance cancer cell adhesion in metastasis of HCC.

## 2. Materials and Methods

### 2.1. Materials

Human normal liver cell line LO-2 and HCC cell lines (HepG2 and BEL-7402) were from Cell Bank National Academy of Science of China (Shanghai, China). Human highly metastatic liver cancer cell line HCCLM3 was from Liver Cancer Institute (Zhongshan Hospital, Fudan University, Shanghai, China). Markers, dNTP, primers were from Shanghai Shenggong Company (Shanghai, China). DMEM medium, RPMI-1640 medium, FBS, Trizol solution, and Lipofectamine 2000 were from Invitrogen Co. (Carlsbad, CA, USA). Reverse transcription kits, restrictive endonuclease* Bgl*II,* Sal*I, and T4 DNA ligase were from MBI Fermentas China Co., Ltd. (Shenzhen, China). BCA protein quantitative kit and real-time qPCR kit (SYBR Green) were from Tiangen Biotech Co., Ltd. (Beijing, china). Plasmid pcDNATM6.2-GW/EmGFP-miR (pmiR) and pcDNATM6.2-GW/EmGFP-miR-Negative Control (pmiR-NC) were from Beinuo Biotech Co. Ltd. (Shanghai, China). PCR purification kits and plasmid extraction kit were from Axygen Scientific Inc. (Carlsbad, CA, USA). Rabbit-anti-HPSE polyclonal antibody and horseradish peroxidase- (HRP-) conjugated anti-rabbit IgG were from Jinqiao Biotechnology (Beijing, China). Rabbit-anti-phosphoglyceraldehyde dehydrogenase (GAPDH) polyclonal antiserum was from Santa Cruz Biotechnology Inc. (Santa Cruz, CA, USA). Matrigel was from BD Bioscience, (San Jose, CA, USA).

### 2.2. Determination of HPSE Expression in HCC Cells

#### 2.2.1. Quantitative Real-Time Reverse Transcription Polymerase Chain Reaction (qRT-PCR)

Human HCC cells and normal liver cell line LO-2 were cultured in RPMI-1640 medium with 10% FBS. According to Gene ID of HPSE mRNA sequence (NM_006665), PCR primers were designed and synthesized (Table 1). The qRT-PCR was performed according to the methods as described in our paper [12], and GAPDH was used as loading control. The experiments were performed for three times.

#### 2.2.2. Western Blotting

Cells were lysed in lysis buffer, incubated on ice for 30 min, and centrifuged for 20 min to remove cell debris. Total cell lysate was subjected to SDS-polyacrylamide gel electrophoresis. The proteins were measured with Western blotting according to the methods as described in our paper [12], and GAPDH was used as loading control. The experiments were performed for three times.

### 2.3. Plasmid Construction and Identification

In the experiment, four HPSE RNAi vectors were constructed using miRNA technique, which is the same as the short hairpin RNA (shRNA) in essence, but an artificial flanking pri-miRNA sequence is extended from the ends of shRNA target sequences. Based on above principle, four pairs of miRNA single-stranded oligonucleotide were designed and synthesized according to Gene ID of HPSE mRNA sequence (Table 2) and then converted into double-stranded form by denaturation and subsequent annealing. The 4 specific double-stranded miRNA sequences were cloned into the pmiR vector, respectively. The products were then transformed into* Escherichia coli* competent cells and cultivated on a plate containing spectinomycin overnight at 37°C. Plasmid DNAs were extracted and sequenced. The samples with correct sequence were named pcDNA-miR-HPSE-1, pcDNA-miR-HPSE-2, pcDNA-miR-HPSE-3, and pcDNA-miR-HPSE-4, respectively.

### 2.4. Transfection and Assessment

Recombinant plasmids pmiR-HPSE-1, pmiR-HPSE-2, pmiR-HPSE-3, and pmiR-HPSE-4 were, respectively, transfected into HCCLM3 cell using Lipofectamine 2000 following the manufacturer's protocol. No plasmid was used in blank control group and pmiR-NC was used as negative control. Transfection efficiency was observed with invert fluorescence microscope 24 h after transfection. Five hundred cells were randomly counted, and the percentage of EGFP-positive cells was calculated. HPSE expressions in transfected cells were measured by real-time RT-PCR and Western blot analysis 48 h later. The experiments were performed for three times. According to the expression levels of HPSE, one miRNA plasmid with best inhibitory effect was chosen for following experiment.

### 2.5. Determination of Cell Invasion, Migration, and Adhesion Abilities

#### 2.5.1. Transwell Invasion and Migration Assay

The experiments were performed as previously described [22]. For invasion assay, 72 hours after transfection, 5 × 10^4^ transfected HCC cells in serum-free RPMI-1640 were seeded into the upper chambers of each well of 24-well plate with insert (8 mm pore size, Millipore, Billerica, MA, USA) coated with Matrigel. For migration assay, the upper chambers were not coated with Matrigel, and cells were seeded after 48-hour transfection. RPMI-1640 containing 10% FBS was placed in the lower chambers as a chemoattractant. After 24 hours of incubation, cells on the upper membrane surface were wiped off, and the cells that invaded across the Matrigel membrane were fixed with paraformaldehyde and stained with crystal violet. The number of invasive cells was then counted (five randomly chosen fields for each membrane) under an invert microscope (200x). Each condition was done in triplicate.

#### 2.5.2. Adhesion Experiment

Matrigel glue (20 mg/L) was added to a 96-well plate at 100 *μ*L per well. The plate was incubated in a Clean Bench overnight. The redundant glue was washed away with appropriate RPMI-1640 medium. HCC cells were transfected for 48 h. Cells were trypsinized, suspended in PBS, counted, and then seeded on the 96-well plate at 5 × 10^4^ per well. The plate was then incubated in the 5% CO_2_ incubator at 37°C for 2 h. After adding 10 *μ*L of MTT solution (5 mg/mL) per well, the cells were continuously cultured for 4 h. Following adding 200 *μ*L DMSO to each well, the plate was gently oscillated at 110 strokes/min for 10 min. The absorbance at 490 nm of the colored solution (*A*_570 nm_) was measured by a microplate reader. Negative control and blank control were both used. Cell adherence rate (%) = (*A*_570 nm_ of experimental group/*A*_570 nm_ of negative control group) × 100%. Each assay was performed in triplicate wells.

### 2.6. Statistical Analysis

All the data are expressed as mean values ± standard deviation (SD). Comparisons among multiple groups were made with a one-way analysis of variance (ANOVA) followed by *q*-test. *P* < 0.05 was used for statistical significance.

## 3. Results

### 3.1. HPSE Expression in HCC Cells

**Figure 1:**
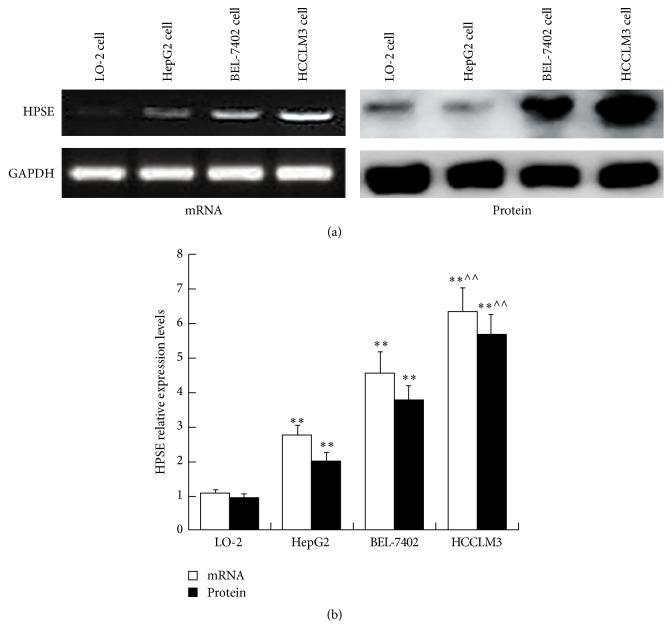
HPSE mRNA and protein expressions in HCC cells. (a) Expressions of HPSE in HCC cells were determined via RT-PCR and Western blot analysis. (b) HPSE mRNA and protein relative expression levels in HCC cells. Data presented means ± SD. ^*∗∗*^ *P* < 0.01 compared with that in LO-2 cell; ^∧∧^ *P* < 0.01 compared with that in HepG2 cell.

HPSE mRNA relative expression levels were higher in HepG2, BEL-7402, and HCCLM3 cells than that in normal hepatocyte (*P* < 0.01). Of all 3 kinds of HCC cells, HPSE showed highest expression level in HCCLM3 cell (*P* < 0.01) (Figure 1). HPSE protein expression was the same as the mRNA expression (Figure 1). According to above results, the HCCLM3 cell was used for subsequent research.

### 3.2. Identification of Recombinant Vectors

**Figure 2:**
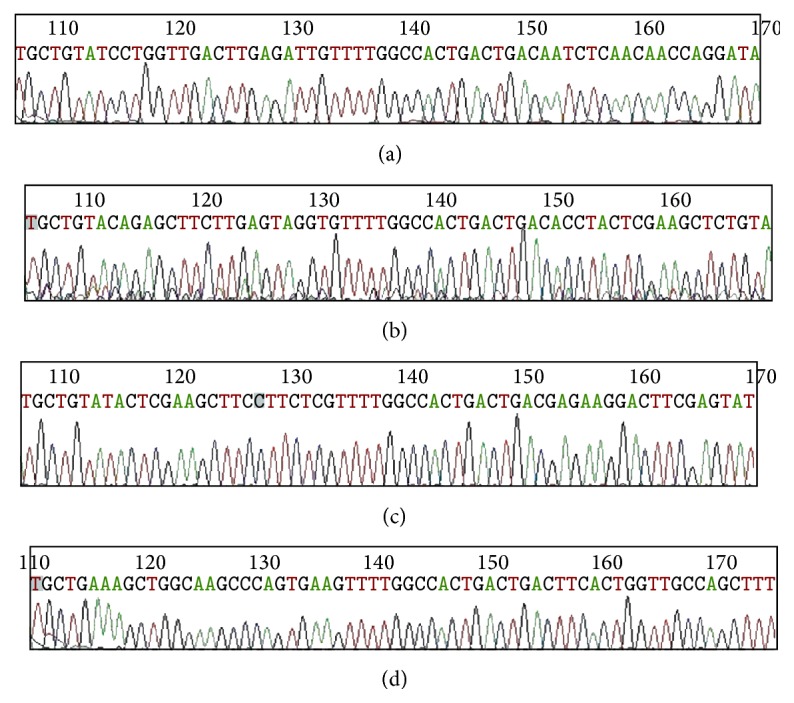
Sequencing graphs of recombinant vectors. ((a)–(d)) Sequencing graphs of 4 target sequences of recombinant vectors pmiR-HPSE-1, pmiR-HPSE-2, pmiR-HPSE-3, and pmiR-HPSE-4, respectively.

The sequencing results showed that all 4 kinds of miRNA vectors were totally consistent with the designing sequence. No deletion, insertion, or mutation was detected (Figure 2). The results suggested HPSE RNAi vector pmiR-HPSE was successfully constructed with miRNA technique.

### 3.3. Transfection Efficiency

**Figure 3:**
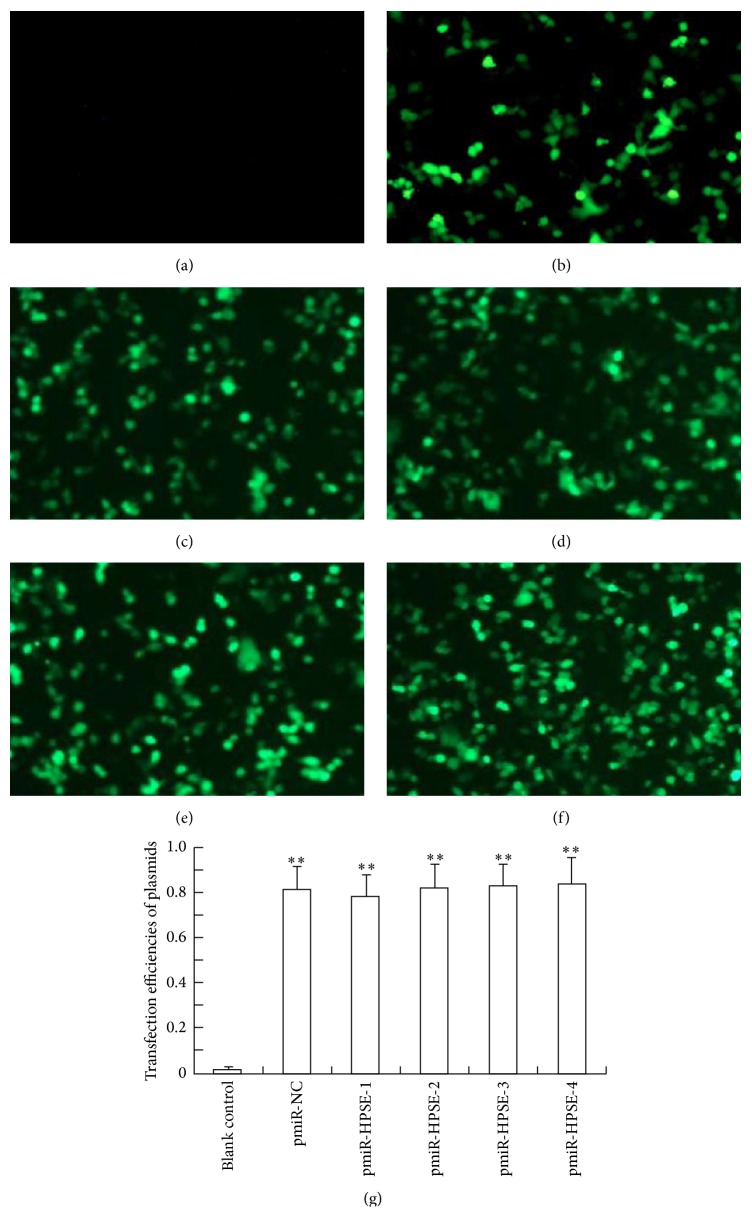
Photofluorograms and transfection efficiencies. (a) No fluorescence could be found in blank control group 48 h later (200x, 48 h); (b) Bright fluorescence could be observed in pmiR-NC group (200x, 48 h). ((c)–(f)) Bright fluorescence in pmiR-HPSE-1/pmiR-HPSE-2/pmiR-HPSE-3/pmiR-HPSE-4 groups (200x, 48 h), respectively. (g) Transfection efficiencies of every group. Data presented means ± SD. ^*∗∗*^ *P* < 0.01 compared with that in blank control group.

After cell transfection, no fluorescence was found in blank control group. Bright fluorescence in negative control or 4 kinds of recombinant plasmid transfected cells could be observed using fluorescence analysis 48 h later. The average transfection efficiencies of negative control and recombinant plasmids ranged from 75% to 85% without significant difference among them (*P* > 0.05) but were all significantly higher than that of blank control group (*P* < 0.01) (Figure 3). These results suggested that recombinant plasmids were successfully transfected into the specific HCC cells.

### 3.4. Effect of Recombinant Plasmids on HPSE Expression in HCC Cells

**Figure 4:**
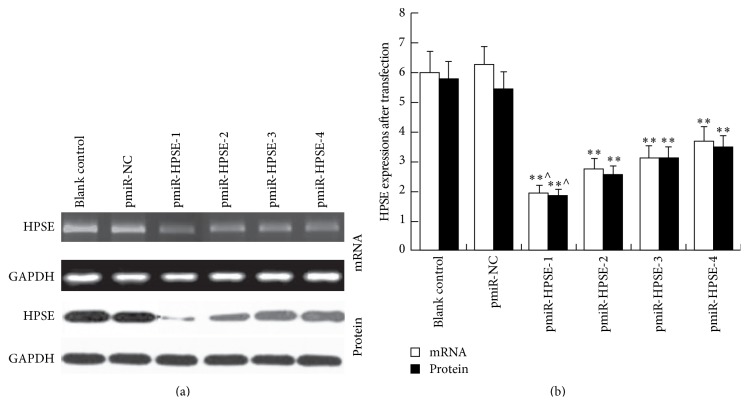
HPSE expressions in pmiR-HPSE transfected HCCLM3 cells. (a) Expressions of HPSE in transfected HCCLM3 cells were determined via RT-PCR and Western blot analysis. (b) Relative expression levels of HPSE mRNA and protein. Data presented means ± SD. HPSE expressions in all pmiR-HPSE groups were significantly lower than those in control groups. ^*∗∗*^ *P* < 0.01 compared with those in control groups. HPSE expression in pmiR-HPSE-1 group was significantly lower than those in pmiR-HPSE-2/pmiR-HPSE-3/pmiR-HPSE-4 groups. ^∧^ *P* < 0.05 compared with those in pmiR-HPSE-2/pmiR-HPSE-3/pmiR-HPSE-4 groups.

Both HPSE mRNA and protein expressions in pmiR-HPSE transfected HCCLM3 cells were significantly lower than those in control groups (*P* < 0.01). There was no obvious difference between blank control and pmiR-NC groups (*P* > 0.05). The maximal decrease was shown in pmiR-HPSE-1 group (*P* < 0.05), and the inhibition ratio approached to 70% (Figure 4). Therefore, plasmid pmiR-HPSE-1 was selected for following invasion and adhesion experiments.

### 3.5. Effect of Plasmid Transfection on Invasion, Migration, and Adhesion Abilities of HCC Cells

**Figure 5:**
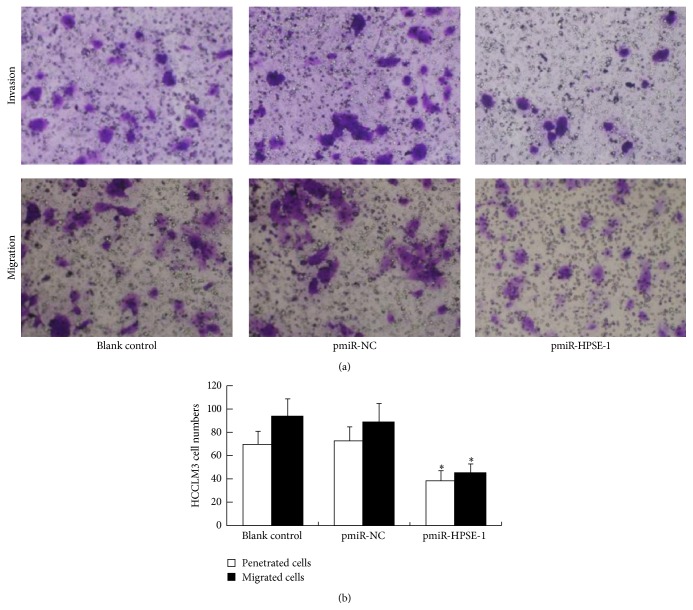
Effect of silencing HPSE gene on invasion and migration ability of HCC cells. (a) Invasion and migration experiments. Penetrated and migrating cells were both decreased after HCCLM3 cells were transfected with pmiR-HPSE-1. (b) Penetrated and migrating HCCLM3 cell numbers. Data presented means ± SD. ^*∗*^ *P* < 0.05 compared with those in control groups.

**Figure 6:**
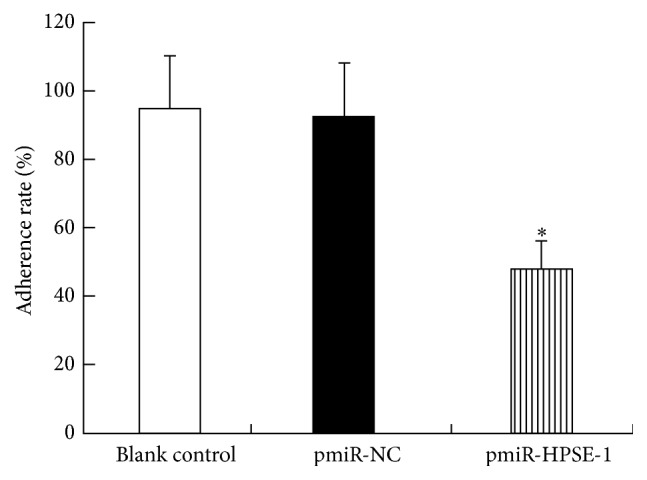
Effect of silencing HPSE gene on adhesion ability of HCC cells. HCCLM3 cell adherence rate significantly decreased after pmiR-HPSE-1 transfection. Data presented means ± SD. ^*∗*^ *P* < 0.05 compared with those in control groups.

The number of penetrated HCCLM3 cells in pmiR-HPSE-1 group was significantly less than those in blank control and pmiR-NC groups (*P* < 0.05). There was no obvious difference between two groups (*P* > 0.05) (Figure 5). Cell migration showed similar results (Figure 5). The adherence rate in pmiR-HPSE-1 group showed significant decrease compared with those in blank control and pmiR-NC groups (*P* < 0.05), and there was no obvious difference between two control groups (*P* > 0.05) (Figure 6).

## 4. Discussion

High levels of HPSE mRNA and protein are expressed in most malignant tumors including HCC and are closely associated with tumor metastasis, angiogenesis, and other diverse pathological processes [8–10]. In this study, we found that both HPSE mRNA and protein expressions in 3 kinds of HCC cells were higher than those in normal hepatocyte, which were similar to previous results [11, 12]. In addition, the HPSE expression levels are different among different HCC cells. Of all 3 kinds of HCC cells, highly metastatic HCCLM3 cells showed highest HPSE expression level. These findings suggested that invasion and metastasis potentials of HCC were positively correlated with HPSE expression level.

HPSE is believed to play an important role in the process of tumor invasion and metastasis [8–15]. In order to verify its prometastasis function, we constructed RNAi vector pmiR-HPSE using miRNA technique. The RNAi based on miRNA context may provide an efficient and safe therapeutic knockdown effect on target gene HPSE [23]. The results proved all 4 recombinant plasmids could significantly decrease HPSE expression in HCCLM3 cells, and pmiR-HPSE-1 showed strongest inhibitory effect. In the following experiment, we demonstrated that pmiR-HPSE-1 can lead to the obvious decrease in the invasion and metastasis capabilities of HCC cells. Therefore, downregulation of HPSE expression could result in suppression of invasion and metastasis abilities of HCC cells, which were similar to other studies [13–15].

On the other hand, HPSE can exert proadhesion or procoagulation activity in hematogenous metastasis and inflammation. HPSE can augment the adhesion of human neutrophils and mononuclear cells to human umbilical vein endothelial cells in a concentration-dependent manner [24], HPSE-miRNA transfection significantly decreases the adhesion ability of melanoma cells besides the invasion and migration abilities [23], and mollusk heparan sulfate inhibits LS180 colon carcinoma cell adhesion [25]. Our adhesion experiment also found pmiR-HPSE-1 significantly attenuated the adherence rate of HCC cells while it obviously inhibited the invasion and migration abilities. Tumor cell shows different adhesion capability in different environment condition. In our adhesion experiment, the plate was gently oscillated at 110 strokes/min after it was incubated. We think it could imitate shaken adhesion or rolling adhesion in blood vessel [26]. Adhered cells in blood vessel possess a protective barrier and escape from immune surveillance resulting in higher capability of invasion and metastasis [27]. Based on above findings and analysis, we conclude that HPSE not only plays an important role in tumor invasion and migration, but also contributes to the adhesion of HCC cells. To our knowledge, this is the first report that HPSE plays a proadhesive role in cell adhesion and tumor microembolus of HCC. There are three possible reasons. The first is HPSE-mediated degradation of HSPGs in ECM of BM. Integrity of BM barrier is destroyed. Second, various vascular growth factor and adhesion molecule were delivered. P-selectin is known to participate in interactions involving tumor cells, platelets, leukocytes, and endothelium, and heparin has been shown to inhibit P-selectin and as a consequence it blunts metastasis and inflammation [25]. Third, HPSE and membrane HSPGs activate signaling molecules such as Akt, Src, epidermal growth factor receptor, and Rac [28]. Of course, further study is needed to explore the concrete mechanism.

## 5. Conclusions

In conclusion, HPSE is a potentiator of cell adhesion in metastasis of HCC.

## Figures and Tables

**Table 1:** Primer sequences for PCR amplification.

Primers	Primer sequences	Length of products (bp)
HPSE-F	5′-GCACAAACACTGACAATCCAAG-3′	101
HPSE-R	5′-AAAAGGATAGGGTAACCGCAA-3′	

GAPDH-F	5′-GTGGTCTCCTCTGACTTCAACA-3′	136
GAPDH-R	5′-CCACCACCCTGTTGCTGTAG-3′	

**Table 2:** miRNA oligo-DNA sequence of human heparanase gene.

Oligonucleotide	Sequences of miRNA oligo	Corresponding sequence of mRNA
pmiR-HPSE-1-F	5′-TGCTGTATCCTGGTTGACTTGAGATTGTTTTGGCCACTGACTGACAATCTCAACAACCAGGATA-3′	356–376 bp
pmiR-HPSE-1-R	5′-CCTGTATCCTGGTTGTTGAGATTGTCAGTCAGTGGCCAAAACAATCTCAAGTCAACCAGGATAC-3′	

pmiR-HPSE-2-F	5′-TGCTGTACAGAGCTTCTTGAGTAGGTGTTTTGGCCACTGACTGACACCTACTCGAAGCTCTGTA-3′	490–570 bp
pmiR-HPSE-2-R	5′-CCTGTACAGAGCTTCGAGTAGGTGTCAGTCAGTGGCCAAAACACCTACTCAAGAAGCTCTGTAC-3′	

pmiR-HPSE-3-F	5′-TGCTGTATACTCGAAGCTTCCTTCTCGTTTTGGCCACTGACTGACGAGAAGGACTTCGAGTATA-3′	1280–1301 bp
pmiR-HPSE-3-R	5′-CCTGTATACTCGAAGTCCTTCTCGTCAGTCAGTGGCCAAAACGAGAAGGAAGCTTCGAGTATAC-3′	

pmiR-HPSE-4-F	5′-TGCTGAAAGCTGGCAAGCCCAGTGAAGTTTTGGCCACTGACTGACTTCACTGGTTGCCAGCTTT-3′	1560–1580 bp
pmiR-HPSE-4-R	5′-CCTGAAAGCTGGCAACCAGTGAAGTCAGTCAGTGGCCAAAACTTCACTGGGCTTGCCAGCTTTC-3′	

## Abbreviations

HCC: Hepatocellular carcinoma
HPSE: Heparanase
HSPG: Heparan sulfate proteoglycan
ECM: Extracellular matrices
BM: Basement membrane
bFGF: Basic fibroblast growth factor
VEGF: Vascular endothelial cell growth factor
RT-PCR: Reverse transcriptase-polymerase chain reaction
pmiR: pcDNATM6.2-GW/EmGFP-miR
pmiR-NC: pcDNATM6.2-GW/EmGFP-miR-Negative Control
GAPDH: Phosphoglyceraldehyde dehydrogenase
shRNA: Short hairpin RNA.
